# A Functional Role for ADAM10 in Human Immunodeficiency Virus Type-1 Replication

**DOI:** 10.1186/1742-4690-8-32

**Published:** 2011-05-11

**Authors:** Brian M Friedrich, James L Murray, Guangyu Li, Jinsong Sheng, Thomas W Hodge, Donald H Rubin, William A O'Brien, Monique R Ferguson

**Affiliations:** 1 Departments of Pathology and Internal Medicine, University of Texas Medical Branch, 301 University Blvd, Galveston, TX, 77555, USA; 2 Zirus, Inc., 1384 Buford Business Boulevard, Suite 700, Buford, GA, 30518, USA; 3 Departments of Medicine and Microbiology & Immunology, Vanderbilt University, 1161 21st Ave South, Nashville, TN, 37232, USA; 4 Research Medicine, VA Tennessee Valley Healthcare System, 1310 24th Ave South, Nashville, TN 37212, USA; 5 Department of Medicine, Division of Infectious Diseases, Emory University School of Medicine, 201 Dowman Dr., Atlanta, GA, 30322, USA

## Abstract

**Background:**

Gene trap insertional mutagenesis was used as a high-throughput approach to discover cellular genes participating in viral infection by screening libraries of cells selected for survival from lytic infection with a variety of viruses. Cells harboring a disrupted *ADAM10 *(A Disintegrin and Metalloprotease 10) allele survived reovirus infection, and subsequently ADAM10 was shown by RNA interference to be important for replication of HIV-1.

**Results:**

Silencing ADAM10 expression with small interfering RNA (siRNA) 48 hours before infection significantly inhibited HIV-1 replication in primary human monocyte-derived macrophages and in CD4^+ ^cell lines. In agreement, ADAM10 over-expression significantly increased HIV-1 replication. ADAM10 down-regulation did not inhibit viral reverse transcription, indicating that viral entry and uncoating are also independent of ADAM10 expression. Integration of HIV-1 cDNA was reduced in ADAM10 down-regulated cells; however, concomitant 2-LTR circle formation was not detected, suggesting that HIV-1 does not enter the nucleus. Further, ADAM10 silencing inhibited downstream reporter gene expression and viral protein translation. Interestingly, we found that while the metalloprotease domain of ADAM10 is not required for HIV-1 replication, ADAM15 and γ-secretase (which proteolytically release the extracellular and intracellular domains of ADAM10 from the plasma membrane, respectively) do support productive infection.

**Conclusions:**

We propose that ADAM10 facilitates replication at the level of nuclear trafficking. Collectively, our data support a model whereby ADAM10 is cleaved by ADAM15 and γ-secretase and that the ADAM10 intracellular domain directly facilitates HIV-1 nuclear trafficking. Thus, ADAM10 represents a novel cellular target class for development of antiretroviral drugs.

## Background

Cell homeostasis and ordered proliferation require the interaction of cellular elements that can be assigned to functional pathways. While cells have partial redundancy and regulated expression of components of important cellular pathways, simple pathogens such as viruses appear to be restricted in their interactions. Based upon the hypothesis that disruption of specific cellular proteins would still allow cell and host survival but restrict or inhibit pathogen replication, we have randomly disrupted cellular genes with an insertional mutagen and selected for candidate genes whose inactivation allows cell survival following lytic infection. Previously, we reported this strategy was successful in the discovery of several critical host genes, including components of the IGF-II pathway for reovirus and Rab9 for Marburg virus, validating the initial hypothesis [1-3]. Moreover, we reasoned that viruses evolved from common ancestral archetypes might exhibit conserved viral-host protein-protein interactions. Thus, we tested whether candidate genes discovered had broad capability to facilitate replication of viruses from other families and found that disruption of the Rab9 pathway also limited the replication of Ebola virus, measles virus, and HIV-1 [1]. HIV-1 replication requires the assistance of multiple host cell functions for productive infection and several participating cellular factors have been identified. Recent large-scale siRNA screens have revealed hundreds of host factors that participate in a broad array of cellular functions and implicate new pathways in the HIV-1 life cycle [4-8]. Host cell encoded factors are required during every step of virus replication, with the possible exception of initiation of reverse transcription [9-12].

We identified A Disintegrin And Metalloprotease 10 (*ADAM10*) in a gene trap library selected for resistance to lytic infection with reovirus and subsequently found that *ADAM10 *expression is critical for HIV-1 replication. ADAM10 is a cellular metalloprotease that activates numerous and diverse cellular proteins via proteolytic cleavage. In addition to its metalloprotease domain, it also contains a disintegrin domain, an EGF-like domain, a cysteine-rich domain, a transmembrane domain, and a cytoplasmic domain [13]. ADAM10 is required in NOTCH signaling during embryogenesis [14]. It also shares some functions with ADAM17 in the cleavage and release of surface bound TNF-α, E-cadherin, and other proteins [15-20]. Previous studies have indicated that ADAM10 is found in both the cellular and nuclear membranes [21,22]. It has been shown that a released intracellular fragment (ICF) of ADAM10 is capable of translocating into the nucleus and is potentially important in the nuclear transport of the androgen receptor [21]. Tousseyn and colleagues have shown that this nuclear entry of ADAM10 is dependent upon sequential proteolytic modification, and demonstrated that the ectodomain of ADAM10 is first shed by either ADAM9 or ADAM15 and the intracellular domain is subsequently cleaved by γ-secretase, releasing the ICF [23].

In studies reported herein, it was found that transfecting cells with ADAM10 small interfering RNA (siRNA) dramatically inhibited replication of X4 and R5 HIV-1 strains, both in primary human monocyte-derived macrophages and in CD4^+ ^cell lines. Moreover, our data indicate that ADAM10 is critical for post-entry HIV-1 replication events occurring during nuclear trafficking or nuclear entry in human monocyte-derived macrophages and in CD4^+ ^cell lines, and is dependent upon its proteolytic modification. Furthermore, we show that ADAM15 and γ-secretase are also required for HIV-1 replication, suggesting that the ADAM10 intracellular domain (ICD) is required for nuclear trafficking of HIV-1 to the nucleus.

## Results

### Implication of ADAM10 in reovirus replication using gene trap insertional mutagenesis

We have applied gene trap insertional mutagenesis [1-3,24] as a high throughput genetic screen to aid in the discovery of novel genes critical for viral replication. Cellular alleles are randomly inactivated, and cells surviving an otherwise toxic viral infection harbor a mutated gene, whose wild type counterpart is potentially utilized in the viral life cycle [1,3]. To identify targets for broad-spectrum viral inhibition, we determined whether candidate genes implicated in gene trap studies with unrelated viruses serve a functional role in HIV-1 replication. Small interfering RNA (siRNA) was used to knockdown expression of candidate genes, and the effect on HIV infection was determined by assaying HIV-1 p24 production. HeLa cells modified to stably express CD4 and CCR5 (TZM-bl cells) were screened with siRNAs targeting genes trapped with reovirus, influenza A, or Marburg virus 48 h prior to infection with LAV (X4-tropic). Treatment of TZM-bl cells with siRNA specific for ADAM10 inhibited HIV-1 replication ~90% (n = 4, data not shown). We also observed that siRNA targeting ERBB2IP did not affect HIV-1 replication, and thus was also used in these studies as a negative control.

### ADAM10 silencing inhibits both R5- and X4-tropic HIV-1 replication

**Figure 1:**
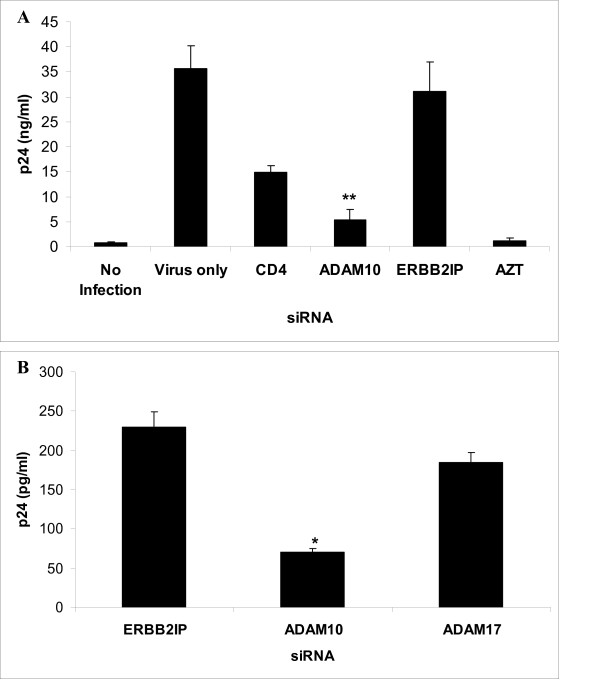
**ADAM10 silencing inhibits both CCR5- and CXCR4-tropic HIV-1 replication**. (**A**) Primary macrophages were transfected with different siRNAs 48 h prior to infection with HIV-SF162. Azidothymidine (AZT) treatment was used as a positive control to block infection. Supernatants were collected 7 days after infection and HIV p24 production was measured by ELISA. ADAM10 silencing significantly reduced viral replication in primary human macrophages (**P < 0.01). (**B**) To determine whether ADAM17 also plays a role in HIV-1 replication, TZM-bl cells were transfected with ADAM17, ADAM10 or ERBB2IP siRNAs 48 h prior to infection with LAV, and supernatant HIV p24 was measured by ELISA 3 days after infection (*P < 0.05).

To confirm the requirement of ADAM10 in more physiologically relevant primary cells, human blood-derived macrophages were transfected with siRNAs targeting ADAM10, CD4, or with a scrambled sequence control siRNA, and then infected with the R5 HIV-1 strain SF162. Figure 1A shows that ADAM10 silencing effectively inhibited R5-tropic HIV-1 replication when human monocyte-derived macrophages were transfected with siRNAs 48 h prior to infection. ADAM10 siRNA inhibition of HIV-1 was similar to that seen with siRNA directed against CD4, the primary cellular receptor for HIV-1 [25].

Interestingly, ADAM10 silencing also inhibited replication of the X4-tropic LAV strain in CD4^+ ^TZM-bl cells (Figure 1B). ADAM17 is a related metalloprotease which shares partial (but not complete) substrate specificity with ADAM10 [15-20], and has been shown to mediate SARS-CoV envelope shedding [26]. Accordingly, the role of ADAM17 expression in HIV-1 replication was studied by knockdown of expression with RNAi in TZM-bl cells; however, ADAM17 silencing did not significantly inhibit viral replication. These results indicate that ADAM10, but not ADAM17, serves a specific role in the viral lifecycle.

### ADAM10 silencing for one week does not affect macrophage viability or function

**Figure 2:**
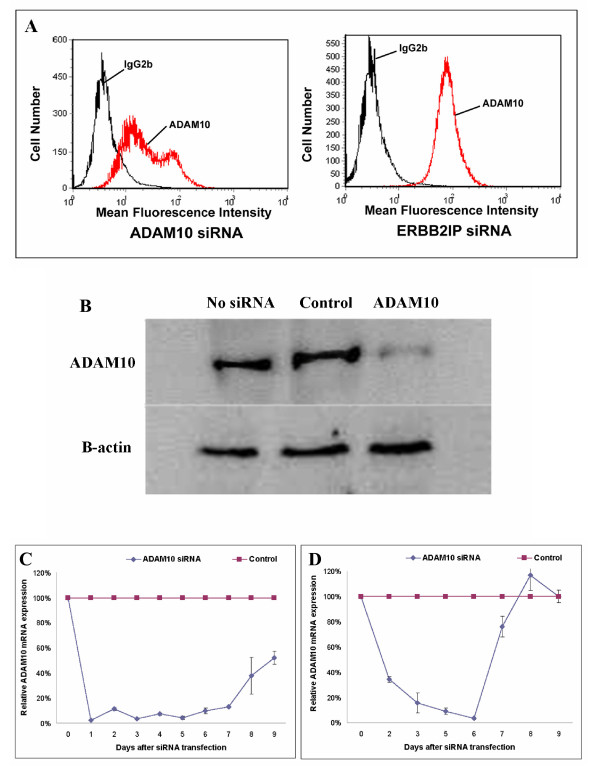
**ADAM10 silencing persists for one week following siRNA transfection**. Assessment of ADAM10 expression following RNAi by (**A**) flow cytometry in TZM-bl cells 48 hours after transfection, (**B**) Western blot in primary human macrophages 48 hours after transfection, and real time PCR in (**C**) U373-MAGI-CCR5 cells and (**D**) primary human macrophages at various times after siRNA transfection. Relative levels of ADAM10 mRNA expression in siRNA transfectants were normalized to GAPDH expression (n = 4).

ADAM10 siRNA transfectants were confirmed to have significant reductions in ADAM10 protein expression by both flow cytometry in cell lines 48 h after siRNA transfection (Figure 2A) and Western blot analysis in primary macrophages 48 h after siRNA transfection (Figure 2B). Kinetics of ADAM10 mRNA down-regulation by siRNA was measured using real time PCR in U373-MAGI-CCR5 cells (Figure 2C) and primary human macrophages (Figure 2D). To determine the viability of siRNA-transfected macrophages, cytotoxicity was assayed using GAPDH coupled to 3-phosphoglyceric phosphokinase and measuring ATP [27]. The siRNA transfections resulted in no significant adverse cytotoxic effect (P < 0.01), although dose-dependent cell death was observed when cells were treated with chelerythrine, a Protein Kinase C inhibitor (data not shown). In addition, ADAM10 siRNA-transfected macrophages displayed phagocytic function similar to macrophages transfected with scrambled siRNA, as determined by comparing the phagocytosis of captured bodipy beads [28] (data not shown).

### ADAM10 expression is not required for HIV-1 reverse transcription

**Figure 3:**
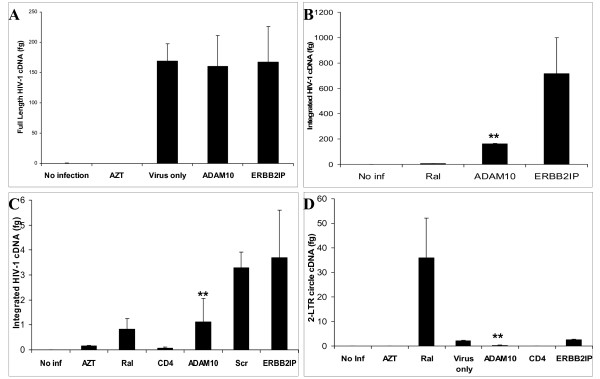
**HIV-1 nuclear entry, but not completion of reverse transcription, is affected by ADAM10 down-regulation**. (**A**) Primary human macrophages were transfected with either ADAM10 or ERBB2IP siRNA 48 h prior to infection with HIV-SF162. DNA was isolated 48 h after infection and real time PCR was used to quantitate formation of full length HIV cDNA. In order to amplify HIV cDNA with these full length primers, two template-switching events and continuous 5'LTR and gag sequences must be present on either strand, which is the last event to occur during HIV reverse transcription [79]. (**B, C**) Integration of HIV was significantly lower in ADAM10 down-regulated (**B**) primary human macrophages and (**C**) U373-MAGI-CCR5 cells than in control ERBB2IP down-regulated cells following infection with HIV-SF162. Genomic DNA was used to quantitate integrated HIV cDNA using real-time PCR using primers specific for integrated HIV cDNA. (**D**) 2-LTR circle formation in ADAM10 down-regulated macrophages was significantly less than that seen in macrophages treated with the integrase inhibitor raltegravir, and was similar to infected but untreated cells. Formation of 2-LTR circles was quantitated by real-time PCR [80]. (No Inf = No Infection, Ral = Raltegravir, Scr = Scrambled siRNA, **P < 0.01).

To determine whether ADAM10 is required for entry or HIV-1 reverse transcription, small non-genomic DNA was isolated from control- and ADAM10 siRNA-transfected macrophages at 48 h post-infection for quantification by real-time PCR. Previous studies demonstrated that kinetics of reverse transcription is slower in macrophages than in lymphoid cells, and full-length HIV-1 reverse transcripts are not generated until 36-48 h after infection in macrophages [29,30]. Thus, cDNA levels detected at 48 hours post-infection should be reflective of only a single replication cycle. As shown in Figure 3A, *ADAM10 *silencing did not affect detection of full length HIV-1 cDNA, whereas viral DNA formation was not detected in cells treated with the reverse transcription inhibitor AZT. These data demonstrate that ADAM10 expression is not required for HIV-1 entry and completion of reverse transcription.

### ADAM10 activity in HIV-1 replication precedes viral integration

To determine if ADAM10 is required for proviral DNA integration, genomic and small non-genomic cDNA was isolated from cells at various time points post-infection. If HIV-1 cDNA enters the nucleus but does not integrate into the host cell chromosome, then the viral cDNA circularizes to form a 2-LTR circle [31,32], which can be quantified using real-time PCR. Integration is quantified by using one primer directed against HIV-1 and another primer directed against *Alu*, a common repetitive sequence found in the human genome. Knockdown of ADAM10 significantly reduces the amount of integrated HIV-1 cDNA in both macrophages (Figure 3B) and U373-MAGI-CCR5 cells (Figure 3C). Small non-genomic DNA was isolated from cells after infection to quantify formation of 2-LTR circles. As shown in Figure 3D, 2-LTR circle formation was not observed in macrophages treated with siRNA to ADAM10, whereas when cells were treated with the integrase inhibitor, Raltegravir, 2-LTR circles are detected [33]. Inhibition of both HIV-1 integration and 2-LTR circle formation by ADAM10 siRNA indicates that while HIV-1 cDNA is efficiently generated, it is not efficiently translocated into the nucleus of ADAM10 down-regulated cells.

### ADAM10 is utilized for steps prior to HIV-1 tat expression

**Figure 4:**
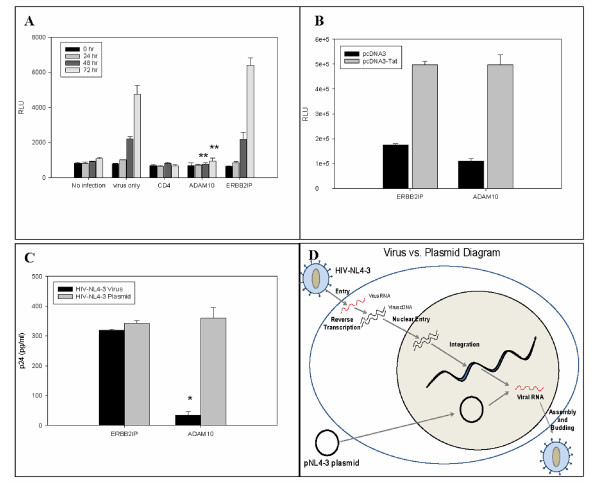
**ADAM10 down-regulation inhibits Tat-dependent HIV-1 replication steps**. (**A**) ADAM10 down-regulation with siRNA affects Tat-dependent β-galactosidase production in U373-MAGI-CCR5 cells after infection with HIV-SF162. β-galactosidase was measured by fluorescence at various time points after infection (**P < 0.01). (**B**) ADAM10 does not directly activate Tat. After either ADAM10 or ERBB2IP down-regulation, U373-MAGI-CCR5 cells were transfected with a plasmid encoding recombinant Tat, indicating that ADAM10 affects virus replication prior to Tat transcription or translation. β-galactosidase is expressed as RLUs (Relative Light Units). (**C**) ADAM10 down-regulation significantly reduces HIV p24 production in virally infected but not HIV plasmid-transfected TZM-bl cells. ADAM10 down-regulation affected viral replication in HIV-NL4-3 infected cells, but not in cells transfected with pNL4-3, a plasmid expressing the full length NL4-3 molecular clone (*P < 0.05) (**D**) Diagram illustrating the different processes for virus production in the virus vs plasmid experiment. Viral mRNA can be directly transcribed from the pNL4-3 plasmid, while virus infection must go through entry, reverse transcription, nuclear entry, and integration to produce viral RNA.

To confirm that ADAM10 function is not required for HIV-1 replication events following integration, U373-MAGI-CCR5 cells were used in reporter gene assays to gauge the effect of ADAM10 silencing on Tat function. Tat function was measured by β-galactosidase (β-gal) activity expressed from a stably integrated HIV-LTR-β-gal construct [34]. As shown in Figure 4A, Tat activity was robust in ERBB2IP control siRNA-transfected and HIV-1 infected, but not transfected (virus only), cells at 72 h. Tat activity in ADAM10 or CD4 siRNA-transfectants at 72 h post-infection was similar to the background levels seen at Day 0. However, Tat activity was unaffected by ADAM10 silencing when U373-MAGI-CCR5 cells were transfected with a plasmid encoding recombinant Tat (Figure 4B), indicating that ADAM10 does not directly activate Tat and that ADAM10 affects virus replication prior to Tat transcription or translation. In agreement, Western blots revealed that production of the viral Env and p24 proteins were significantly inhibited between days 4-7 post-infection in primary macrophages following ADAM10 silencing (data not shown).

Additionally, the role of ADAM10 was studied in TZM-bl cells transfected with a plasmid-based molecular clone (pNL4-3) or infected with the corresponding HIV-NL4-3 virus. Although replication of HIV-NL4-3 was dramatically inhibited in ADAM10 siRNA-transfectants, ADAM10 was not required in the plasmid-based system (Figure 4C). The pNL4-3 plasmid has a 15 kb insert that includes a full-length proviral clone and one to two kb of flanking cellular sequence outside both the 5' and 3' LTR and very efficiently directs HIV gene expression following transfection, independent of plasmid integration. Thus, the plasmid serves as a surrogate for proviral integration, bypassing the normal early events of the viral life cycle (Figure 4D). Additionally, we used a U1 cell line, which contains two integrated copies of the HIV-1 proviral genome, and can be induced to produce progeny virus following treatment with a phorbol ester [35,36]. Down-regulation of ADAM10 had no effect on production of HIV-1 in these cells (data not shown). These data indicate that ADAM10 supports virus replication prior to gene transcription. Taken together, these data suggest that the function of ADAM10 in HIV-1 replication is bracketed between the levels of nuclear trafficking and nuclear entry.

### A functional ADAM10 metalloprotease is not required for HIV-1 replication

**Figure 5:**
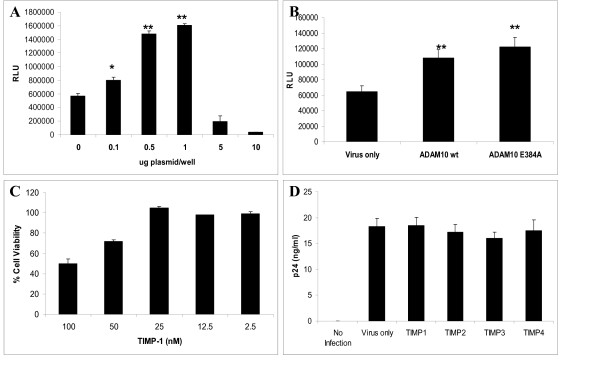
**A functional ADAM10 metalloprotease domain is not required for HIV-1 replication**. (**A**) Over-expression of wt ADAM10 in U373-MAGI-CCR5 cells increased HIV-1 replication; however, cell toxicity was noted at concentrations of 0.5 μg and above. β-galactosidase activity was measured 48 h after infection (*P < 0.05, **P < 0.01). (**B**) Over-expression of wt ADAM10 and ADAM10 E384A both increased HIV-1 replication U373-MAGI-CCR5 cells. 0.5 μg of DNA plasmid was transfected into U373 cells and infected with HIV-SF162 48 hours after transfection. Cells were lysed and β-galactosidase activity was measured 48 h after infection (*P < 0.05). (**C**) Serial dilutions of tissue inhibitors of metalloprotease 1 (TIMP-1) were added to primary macrophages, and cell viability was assessed 24 h after addition of TIMP-1. (**D**) TIMPs had no effect on HIV-1 replication in primary human macrophages. TIMPs (25 nM) were added to primary macrophages 24 h prior to and during infection with HIV-SF162. Supernatant was collected 7 d after infection and HIV-1 p24 production was measured by ELISA.

To determine whether over-expression of ADAM10 increases HIV-1 replication and infection, we obtained a human ADAM10 plasmid from Dr. Stefan Lichtenthaler (LMU Munich, Germany). As shown in Figure 5A, over-expression of ADAM10 resulted in increased HIV-1 replication. The metalloprotease domain potentially responsible for this increase was further investigated. ADAM10 E384A plasmid contains a single inactivating point mutation in the metalloprotease domain rendering the metalloprotease domain inactive [37]. ADAM10 E384A and wild type (wt) ADAM10 plasmids were transfected into U373-MAGI-CCR5 cells 48 h prior to infection with HIV-SF162 (MOI = 0.1). As shown in Figure 5B, over-expression of ADAM10 E384A showed an increase in HIV-1 replication very similar to that seen with wt ADAM10 over-expression, suggesting that the metalloprotease domain is not the critical domain in ADAM10 supporting HIV-1 infection. In addition, tissue inhibitors of metalloproteases (TIMPs) 1 and 3, which have been shown to inhibit ADAM10 metalloprotease activity [38], had no effect on HIV-1 replication in human macrophages (Figure 5C, D). This indicates that the ADAM10 metalloprotease domain is not functionally required for HIV-1 replication.

### Both γ-secretase and ADAM15 are required for HIV-1 replication

**Figure 6:**
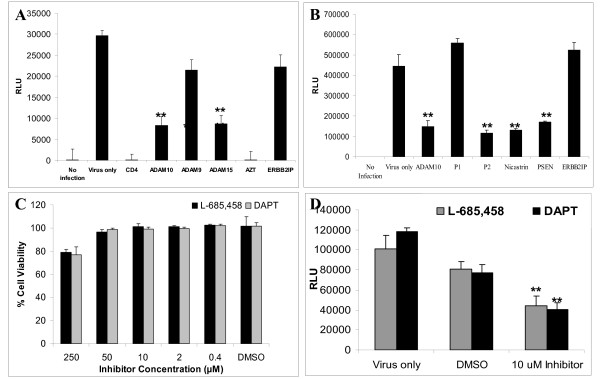
**ADAM15 and γ-secretase are required for HIV-1 replication**. (**A**) ADAM15 downregulation significantly reduced HIV-1 replication in U373-MAGI-CCR5. However, ADAM9 downregulation did not affect HIV-1 replication. U373 cells were transfected with siRNA and β-galactosidase was measured 48 h after infection with HIV-SF162. (**B**) Down-regulation of γ-secretase subunits significantly decreased HIV-1 replication. Presenilin-2, not presenilin-1, is specifically required for HIV-1 replication (P1 = presenilin-1, P2 = presenilin-2, PSEN = presenilin enhancer protein 2, **P < 0.01). β-galactosidase was measured 48 h after infection with HIV-SF162. (**C**) Serial dilutions of γ-secretase inhibitors (L-685,458 and DAPT) were added to the cells, and cell viability was assessed 24 h after addition of inhibitors. (**D**) L-685,458 and DAPT significantly reduced β-galactosidase in U373-MAGI-CCR5 cells compared to DMSO controls. β-galactosidase activity was measured 48 h after infection with HIV-SF162 (**P < 0.01).

To determine if the intracellular domain of ADAM10 plays a role in HIV-1 replication, we independently inhibited the two necessary proteolytic steps that free this fragment. ADAM9 and ADAM15 were shown to cleave the ectodomain of ADAM10 while γ-secretase has been shown to cleave and release the ADAM10 intracellular domain (ICD) [23]. Once released, the ADAM10 ICD can then translocate to the nucleus or peri-nuclear region [21,23]. To determine whether ADAM9 and/or ADAM15 were required for HIV-1 replication, cells were transfected with siRNAs directed against either ADAM9 or ADAM15 mRNA prior to infection. As shown in Figure 6A, ADAM15 siRNA significantly reduced HIV-1 replication, comparable to the level of replication seen with knockdown of ADAM10, whereas ADAM9 knockdown had no effect on HIV-1 replication. Next, we studied the role of γ-secretase, a multi-subunit complex, containing presenilin, nicastrin, anterior pharynx-defective 1 (APH-1), and presenilin enhancer protein 2 (PSEN) [39], in HIV-1 replication. γ-secretase contains either the presenilin-1 (P1) or presenilin-2 (P2) isoform, which contributes to the substrate specificity of the enzyme [40]. To determine if γ-secretase is required for HIV-1 replication, siRNA targeting different components of γ-secretase was used to inhibit the enzyme. As shown in Figure 6B, siRNA targeting P2, nicastrin, and PSEN all significantly decreased HIV-1 replication in U373 cells. However, P1 siRNA did not affect HIV-1 replication. These data show a specific role of presenilin-2, and not presenilin-1, in HIV-1 replication. Additionally, we used specific γ-secretase inhibitors, L-685,458 and DAPT [41,42]. Cytotoxicity assays were performed to determine optimal, sub-toxic concentrations for either inhibitor (Figure 6C). Figure 6D shows that adding 10 μM of either L-685,458 or DAPT to U373 cells 24 h prior to infection and during infection, significantly decreased HIV-1 replication as compared to DMSO-only treatment and infection only controls. These findings confirm that γ-secretase is required for HIV-1 replication. Taken together, both ADAM15 and γ-secretase facilitate HIV-1 replication, consistent with their roles in the release of the ADAM10 intracellular domain.

## Discussion

We utilized gene-entrapment of diploid cell lines for our initial selection of candidate genes associated with cell survival following lytic virus selection. Several possible outcomes may result, including haploid insufficiency, complete loss of expression from a vector inserted into a dominant allele [43-45], or dominant negative effects due to truncated translational products [46,47]. Furthermore, siRNA can be used as a confirmatory step across a wide variety of cell types and viruses, once a candidate gene is identified, as we reported for HIV-1 infection [1]. In this study, we identified *ADAM10 *by gene trap insertional mutagenesis as a disrupted gene in cells surviving cytolytic reovirus infection, and we demonstrated the importance of ADAM10 expression at a post-entry step in HIV-1 replication. We also show that over-expression of ADAM10 increases HIV-1 replication. Interestingly, in previous studies solely using siRNA or shRNA to identify cellular proteins required for HIV-1 replication [4-7], Brass *et al*. had also identified ADAM10 as a required cellular gene [4]. Importantly, these studies show that ADAM10 silencing inhibits HIV-1 in primary human macrophages, which are more relevant to human disease than tissue culture adapted cell lines [48]. Macrophages and CD4^+ ^lymphocytes are the predominant cell types infected with HIV-1 clinically, and the importance of ADAM10 in HIV-1 replication in primary human macrophages supports a role of ADAM10 in HIV-1 pathogenesis.

To determine the precise step in HIV-1 replication in which ADAM10 participates, we inhibited various processes and queried for viral products that define steps up to and including virus expression from chromosomally integrated viral DNA. Our data supports a role for ADAM10 at a step in virus replication prior to integration. HIV-1 must enter the cell and be partially disassembled prior to reverse transcription of viral cDNA, and these steps are not inhibited with knockdown of ADAM10. It was found that ADAM10 silencing resulted in a failure of viral cDNA to integrate, as measured by real time PCR. Moreover, HIV-1 2-LTR circles did not accumulate in the nucleus, which occurs after virus enters the nuclear membrane but cannot integrate. It is known that 2-LTR circles accumulate when integration is inhibited with specific integrase enzyme inhibitors [33]. Furthermore, ADAM10 silencing did not affect Tat-dependent proviral gene expression as assessed in studies using a plasmid expressing Tat and Tat-dependent β-galactosidase expression. Using pNL4-3, a plasmid containing the HIV-1 genome, knockdown of ADAM10 did not limit virus transcription, consistent with its role prior to viral transcription from integrated proviral DNA. Our data are supportive of an important role for ADAM10 in HIV-1 replication at a step following reverse transcription but prior to HIV-1 integration, likely at the level of nuclear trafficking.

A role for ADAM10 during nuclear trafficking is in concert with known cellular roles for this complex protein. The protein has several known extracellular domains, which include a metalloprotease domain, an integrin binding domain, and a cysteine rich region. A recent study has shown ADAM10 to be essential for cell entry of Plasmodium falciparum due to its interaction with the malaria PfSUB2 enzyme [49]. ADAMs function in the proteolytic release of many transmembrane cell surface cytokines, growth factors, receptors, and adhesion proteins, a process known as ectodomain shedding. ADAM10 is known to cleave over 20 cell surface proteins [15-20,50-68]. Most known ADAM10 substrates are involved in cellular adhesion, including ephrin-A2 (EFNA2), AXL, fractalkine (CX3CL1), CXCL16, E- and N-cadherin (CHD1 and 2), the γ-protocadherins C3 and B4, NCAM, CHL1, LAG-3, CD23, CD44, CD46, and desmoglein-2 (DSG2). However, while there is known promotion of trans-infection of HIV-1 secondary to interaction with the adhesion molecules, C-type lectins DC-SIGN and DC-SIGNR [69,70], the data presented above do not support a role in cell entry for ADAM10. Surprisingly, the metalloprotease function was not required for HIV-1 replication. More recently, activity has been attributed to the 6 kDa fragment released from the carboxy-terminus. This fragment is released from the intracellular domain following sequential proteolytic digestion. ADAM9 and -15 have been shown to be responsible for releasing the ADAM10 ectodomain, while presenilin/γ-secretase has been shown to be responsible for the proteolytic release of the ADAM10 intracellular domain from the plasma membrane, whereupon it localizes to the nucleus [23]. Cleavage and release of the ADAM10 ectodomain are required for the intracellular domain to be subsequently released. We demonstrate that both ADAM15 and γ-secretase are required for HIV-1 replication, which strongly suggests the intracellular domain of ADAM10 is critical for HIV-1 replication. We did not find ADAM9 to be required for HIV-1 replication in our assays. Whether this is unique to the cell line used in our assays, as ADAM10 can be alternatively spliced, or rather that ADAM15 is specifically required by HIV-1, requires further study.

## Conclusions

**Figure 7:**
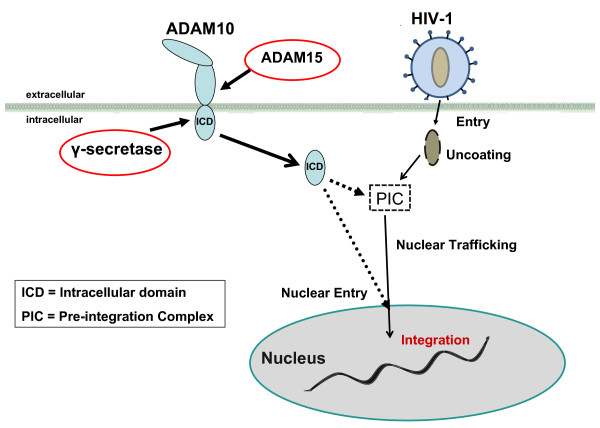
**Hypothesized role for ADAM10 during HIV-1 replication, affecting nuclear trafficking**. ADAM15 cleaves the ADAM10 extracellular domain, followed by cleavage of the ADAM10 intracellular domain (ICD) by γ-secretase. Data presented in this study support a model whereby release of the ADAM10 ICD from the plasma membrane facilitates HIV-1 replication, either by promoting trafficking/docking of the HIV-1 pre-integration complex (PIC) to the nuclear membrane, or PIC translocation into the nucleus.

ADAM10 has a role in androgen receptor nuclear translocation and has been shown to translocate to the nuclear and the perinuclear region during prostate cancer pathogenesis and progression [21]. Combined with our data showing that ADAM10 functions during nuclear trafficking or nuclear entry, we suggest that the intracellular domain may either function to promote trafficking of HIV-1 PIC to the nucleus (Figure 7), or serve a scaffolding role during PIC assembly. The relationship between ADAM10 intracellular domain and HIV-1 PIC needs further study. It is possible that the ICD directly interacts with HIV-1 viral proteins or nucleic acid, or it is essential for another host component that traffics the PIC through the nuclear pore. Studies are ongoing to determine its precise role in HIV-1 entry into the nucleus. It is intriguing to note that ADAM10 is not the only ADAM protein that has dual functionality, as Cousin *et al*. have found that the nuclear translocation of the ADAM13 intracellular domain is required for gene expression and neural crest cell migration [71]. Whether this class of proteins participates in the replicative cycle for other virus may deserve further study.

These studies utilized both primary human macrophages and tissue culture adapted cell lines that have been extensively used in the study of HIV-1. Primary macrophages with knockdown of ADAM10 were viable and functionally active, thereby raising the possibility that inhibition of ADAM10 processing or targeting the intracellular fragment could lead to new set of potential therapeutic targets.

## Methods

### Cells, viruses, and reagents

Monocyte-derived human macrophages were prepared from leukopaks obtained from the University of Texas Medical Branch Blood Bank (Galveston, TX). Peripheral blood mononuclear cells were recovered from leukopaks by Ficoll-Hypaque density centrifugation and were purified by adherence to plastic, as previously described [72]. The following cell lines were obtained from the NIH AIDS Research and Reference Reagent Program, Division of AIDS, NIAID, NIH: CD4/CCR5/CXCR4^+ ^TZM-bl HeLa cells from Dr. John C. Kappes, Dr. Xiaoyun Wu and Tranzyme Inc. [73]; U373-MAGI-CCR5 cells (contributed by Dr. Michael Emerman and Dr. Adam Geballe), are a cell line derived from a glioblastoma that has been modified by stable transfection of LTR-β-galactosidase which is trans-activated by HIV Tat in relation to the level of virus replication [74]. U373-MAGI-CCR5 cells also express CD4 and human chemokine receptor CCR5 to enable infection by HIV R5 strains and were maintained in Dulbecco's modified Eagle's medium (DMEM) supplemented with 10% FBS, 0.2 mg/mL G418, 0.1 mg/mL hygromycin, and 1.0 μg/mL puromycin [75]. Rat intestinal epithelial 1 (RIE-1) cells were maintained in DMEM supplemented with 10% FBS, penicillin, and streptomycin. Primary R5 viruses HIV-SF162 [76] and HIV-SX [72] were purchased from the Virology Core Facility, Center for AIDS Research at Baylor College of Medicine, Houston, TX. HIV-SX stock containing 69.681 ng/ml of HIV p24 with 6.5 × 10^4 ^TCID_50_/ml and HIV-SF162 stock containing 169 ng/ml of HIV p24 with 4.2 × 10^5 ^TCID_50_/ml were used for macrophage and U373 infection experiments at an MOI of 0.1. The following reagents were obtained through the AIDS Research and Reference Reagent Program, Division of AIDS, NIAID, NIH: pNL4-3 from Dr. Malcolm Martin [77] and HIV-LAV [78]. TZM-bl cells were transfected with pNL4-3 plasmid to produce infectious NL4-3 virus, an X4 HIV-1 strain. TIMPs were purchased from R&D Systems, Inc. (Minneapolis, MN 55413). L-685,458 and DAPT were purchased from Sigma-Aldrich (St. Louis, MO).

### siRNA transfections

siRNA SMARTpools targeting candidate host mRNAs were synthesized by Dharmacon (Lafayette, CO). Individual siRNA duplexes targeting ADAM10 mRNA were against positions 1119-1138 (5' CCCAAAGTCTCTCACATTA-3'), 1272-1280 (5'-GGACAAACTTAACAACAAT-3'), 1591-1609 (GCAAGGGAAGGAATATGTA-3'), and 2070-2088 (5'-GCTAATGGCTGGATTTATT-3'). TZM-bl and U373 cells were transfected with Lipofectamine 2000 (Invitrogen), using 50 nM siRNA 48 h before viral infections or harvesting for real time quantitative PCR gene target silencing studies. All macrophage transfections were performed with Oligofectamine (Invitrogen) using 50 nM siRNA 48 h prior to infection following the manufacturer's recommendations, unless otherwise noted.

### Real time quantitative PCR

Primers and probes for real time PCR were custom ordered from Sigma-Aldrich. Full genomic DNA was isolated from monocyte-derived macrophages or cell lines using the Qiagen Blood and Tissue Kit. Small non-genomic DNA, such as reverse transcribed viral cDNA and 2-LTR circles were isolated using a Qiagen Miniprep kit. To detect full length HIV DNA, primers M667 (5'-GGC TAA CTA GGG AAC CCA CTG-3') and M661 (5'-CCT GCG TCG AGA GAG CTC CTC TGG-3') [79], along with probe MH603 (5'-(FAM)-ACA CTA CTT GAA GCA CTC AAG GCA AGC TTT-(TAMRA)-3') [80] were used. The primers used to amplify HIV-1 cDNA span the primer binding site (PBS); the only time there is contiguous DNA on both sides of the PBS is when synthesis of both full strands of viral DNA is completed [29,79]. To identify 2-LTR circle formation, primers MH535 (5'-AAC TAG GGA ACC CAC TGC TTA AG-3') and MH536 (5'-TCC ACA GAT CAA GGA TAT CTT GTC-3') were used with the MH603 probe [80]. For integration, a two-step PCR reaction was performed. For the initial PCR amplification, Alu forward, (5'-GCC TCC CAA AGT GCT GGG ATT ACA G-3'); and HIV-1 gag reverse, (5'-GCT CTC GCA CCC ATC TCT CTC C-3') primers were used [81]. The second step real-time PCR used the primers LTR forward, (5'-GCC TCA ATA AAG CTT GCC TTG A-3'); and LTR reverse, (5'-TCC ACA CTG ACT AAA AGG GTC TGA-3'); and LTR probe (5'-FAM-GCG AGT GCC CGT CTG TTG TGT GAC TCT GGT AAC TAG CTC GC-DBH1-3') [81]. Serial dilutions of purified HIV-SX plasmids were used to generate a standard curve to calculate the total concentration of HIV-1 cDNA in each sample, which is expressed as total fentograms (fg) of cDNA. Total mRNA was isolated from siRNA-transfected cells and primary human macrophages using RNeasy Mini Kits (Qiagen, Inc., Valencia, CA). ADAM10 specific primers and probe were purchased from Applied Biosytems (Carlsbad, CA). All reactions were performed using Applied Biosystems TaqMan Universal Master Mix and run using an Applied Biosystems 7500 Fast Real Time PCR system and 7500 Fast System Software [33]. Silencing of target genes was determined by normalizing target gene expression to GAPDH expression (n = 3).

### Flow cytometry

ADAM10 protein expression was assessed using an analytical flow cytometer in control or ADAM10 siRNA-transfected TZM -bl cells after 48 h. ADAM10 expression in siRNA transfectants was determined by detaching cells in PBS/2 mM EDTA and staining cells using either a mouse anti-human ADAM10 antibody (R&D Systems, Minneapolis, MN) or an IgG1 isotype control (Southern Biotech, Birmingham, AL), followed by incubation with a phycoerythrin-conjugated rabbit anti-mouse secondary antibody (Jackson ImmunoResearch, West Grove, PA).

### HIV-1 p24 assays

To determine the effect of silencing cellular genes on HIV-1 replication, TZM-bl cells or primary human macrophages were transfected with siRNA SMARTpools for 48 h prior to infection. TZM-bl cells were seeded into T25 flasks (750,000 cells/flask) and transfected as described above. Subsequently, cells were infected overnight with HIV LAV (MOI = 1) and then seeded into duplicate T75 flasks (1.5 million cells per flask). p24 production was assayed from culture supernatants 3 days post-inoculation using the p24 antigen ELISA system (Beckman/Coulter/Immunotech, Brea, CA) following the manufacturer's protocol. Macrophages were infected in 24-well plates with HIV-SF162, and p24 production was assayed from cell lysates and culture supernatants 4 and 7 days, respectively, post-infection using p24 Capture ELISA (ImmunoDiagnostics, Woburn, MA).

### β-galactosidase assays

U373-MAGI-CCR5 cells were transfected with ADAM10 or control siRNAs for 48 h, and Tat was expressed by either infection with HIV-SF162 or transfection with a pcDNA3-HIV Tat101-Flag vector (NIH Research and Reference Reagent Program) overnight. At various time points following infection or transfection, cells were lysed and analyzed for β-galactosidase activity using the Beta-Glo Assay System (Promega, Madison, WI) and a Bio-Tek Clarity Microplate Luminometer (pcDNA3-HIV Tat101 Flag transfections) or Dynex MLX Luminometer (SF162 infections).

### DNA plasmid transfections

TZM-bl cells were seeded in 24 well plates at 2 × 10^4 ^cells/well, and transfected with 800 ng HIV-SX plasmid DNA using Lipofectamine LTX transfection reagent (Invitrogen) along with Plus reagent (Invitrogen) using the one tube protocol according to manufacturer's specifications. After three days incubation, p24 was measured by using a p24 Capture ELISA (see above).

Transfection of ADAM10 plasmids (wild type and E384A) was performed using Lipofectamine 2000 (Invitrogen) according to manufacturer's protocols. ADAM10 E384A has a point mutation in its metalloprotease domain, rendering it inactive. U373-MAGI-CCR5 cells were plated in 12-well plates. After plasmid transfection, cells were incubated for 48 h and were then infected with HIV-SF162 (MOI = 0.1). Forty-eight hours after infection, a β-galactosidase assay was used to measure infection in the cells as described above.

### Cell toxicity assays

The toxicity of siRNA treatment was measured by aCella-Tox bioluminescence Cytotoxicity Assay (Cell Technology Inc, Mountain View, CA), which detects secreted Glyceraldehyde-3-Phosphate Dehydrogenase (GAPDH) in cells with diminished membrane integrity. Values for released GAPDH were normalized to cellular GAPDH levels.

### Statistics

All statistics were performed using a two-tailed, paired Student's T-Test (*P < 0.05, **P < 0.01).

## Competing Interests

D.H.R. is the scientific founder of Zirus, Inc., and J.L.M., D.H.R., T.W.H., and W.A.O. have equity in the company. Zirus, Inc. holds intellectual patents to ADAM10.

All other authors declare no competing interests.

## Authors' contributions

BF performed the macrophage and U373 cell experiments and drafted the manuscript. JLM performed the TZM-bl experiments, helped draft the manuscript, and participated in the design of the study. GL helped with the macrophage experiments. JS performed gene trap studies implicating ADAM10 in reovirus replication. TWH, DHR, WAO, and MRF all participated in the design and coordination of the study and helped draft the manuscript. All authors read and approved the final manuscript.
